# Phage Displayed Domain Antibodies (dAb) for Detection of Allergenic Pistachio Proteins in Foods

**DOI:** 10.3390/foods9091230

**Published:** 2020-09-03

**Authors:** Raquel Madrid, Aina García-García, Isabel González, Rosario Martín, Teresa García

**Affiliations:** Departamento de Nutrición y Ciencia de los Alimentos, Facultad de Veterinaria, Universidad Complutense de Madrid, 28040 Madrid, Spain; raqmad01@ucm.es (R.M.); ainagarcia@ucm.es (A.G.-G.); gonzalzi@ucm.es (I.G.); rmartins@ucm.es (R.M.)

**Keywords:** phage-ELISA, 11S globulin, food analysis, domain antibody (dAb), recombinant antibody, pistachio detection, food allergen detection

## Abstract

Pistachio nuts (*Pistacia vera*) have been consumed by past and present-day civilizations because of their organoleptic characteristics and potential health benefits. However, they can also produce moderate to severe IgE-mediated reactions in allergic individuals. In this work, we report the isolation of the first recombinant antibodies against pistachio nut, produced without animal immunization, to be used in immunoassays for detection of allergenic pistachio in food products. Several phage display biopanning strategies were evaluated to screen the human-based domain antibody library (dAb) in search for pistachio-specific probes. The clone producing the PVF4 phage-dAb was finally selected, and it does not cross-react with cashew despite the phylogenetic proximity with pistachio. Western blot and matrix-assisted laser desorption/ionization tandem mass spectrometry (MALDI-TOF/TOF) analysis demonstrated that this clone recognised a unique band of ∼22 kDa related to the basic subunit of pistachio 11S globulin (allergen Pis v 2). The PVF4 phage-dAb allowed detection of pistachio in a food matrix with a limit of detection (LOD) of 3983 mg kg^−1^ in an indirect phage-enzyme-linked immunosorbent assay (ELISA). The ELISA method developed was used to assess applicability of the PVF4 phage-dAb for analysis of 77 commercial food products.

## 1. Introduction

Pistachio nuts (*Pistacia vera*) have been part of the human diet since prehistoric times and have been consumed because of their organoleptic characteristics and potential health benefits [1]. However, they can also produce moderate to severe IgE-mediated reactions in allergic individuals. Moreover, pistachio-allergic patients often report allergy to cashews, probably due to the close relation of these two members of the *Anacardiaceae* family [2,3]. Until now, the allergen nomenclature sub-committee of the World Health Organization and International Union of Immunological Societies (WHO/IUIS) has recognised five allergenic proteins for pistachio nut, named Pis v 1, Pis v 2, Pis v 3, Pis v 4, and Pis v 5 [4,5,6].

Pistachio-allergic patients should thoroughly read product labelling, understand it, and avoid products with allergen warnings [7]. Moreover, to ensure compliance with food labelling regulations and provide accurate information to allergic consumers, it is key to establish an effective risk assessment of allergens in the food industry, including assessment of undeclared allergens in raw materials purchased from suppliers. This requires rapid, specific, and sensitive methods to detect allergens in raw and processed foods [8].

There are several genetic and immunological methods available for the detection of pistachio in food products [9,10,11]. Immunoassays are the most widely used methods for the detection of pistachio in foods, and there are some commercial kits available. However, all the immunoassays currently available for this purpose rely on the use of polyclonal or monoclonal antibodies raised in animals. Current international regulations on animal welfare (European Directive 2010/63/EU) firmly encourage the development of alternatives based on the principle of the ‘Three Rs’: to replace, reduce, and refine the use of animals in research and testing procedures [12]. Therefore, there is a need for alternatives to the use of experimental animals to obtain antibodies capable to detect pistachio allergens in food products. The development of immunoassays based on recombinant antibodies that do not depend on in vivo immunizations is still incipient and provides a novel and promising alternative for the detection of food allergens.

Using phage display technology, recombinant antibodies of defined specificity and constant amino acid sequence can be produced without animal immunization for use in immunoassays. This method uses libraries of recombinant phage antibodies that display functional antibody fragments, like single-chain variable fragments (scFv) or heavy chain variable domains (VH), in their surface. The application of phage display technology for the detection of food allergens has significant potential, but it is still limited to the detection of some allergenic tree nuts with recombinant scFvs [13,14]. Compared to other antibody fragments, like scFv and Fab, VH single domain antibodies or nanobodies have a smaller size (14 kDa), higher solubility and stability, and excellent tissue penetration in vivo. Moreover, they can be genetically linked or chemically conjugated to different molecules to facilitate their use in immunoassays or as therapeutic agents [15]. Isolation of phage-antibody fragments of the desired specificity is achieved by an iterative biopanning procedure with the immobilised antigen [16].

In this work, we report the isolation of recombinant antibodies against pistachio nut from the human based domain antibody library (dAb) by an iterative affinity selection procedure, avoiding animal immunization. We also describe an indirect phage-enzyme-linked immunosorbent assay (ELISA) that allows for the detection of pistachio in commercial food products.

## 2. Materials and Methods

### 2.1. Materials and Chemicals

The protein extraction buffer consisted of 0.035 M phosphate solution containing 1 M NaCl, pH 7.5. Phosphate-buffered saline (PBS) composition is 0.01 M phosphate buffer, 0.0027 M potassium chloride, and 0.137 M sodium chloride, pH 7.4. Milk phosphate-buffered saline (MPBS) contains 1% skimmed milk powder in PBS. PBST is PBS containing 0.01% Tween 20. Tryptone, yeast extract and European bacteriological agar were purchased from Laboratorios Conda (Madrid, Spain). The 2 × TY broth is 16 g L^−1^ tryptone, 10 g L^−1^ yeast extract, and 5 g L^−1^ NaCl. TYE agar is 15 g L^−1^ bacto-agar, 10 g L^−1^ tryptone, 5 g L^−1^ yeast extract and 8 g L^−1^ NaCl. Sample buffer is 0.5 M Tris-HCl buffer, pH 6.8, 10% SDS, 20% glycerol, 0.5% bromophenol blue as the tracking dye, and 5% β-mercaptoethanol. BlueSafe to stain proteins in an SDS-PAGE was provided by NZytech (Lisbon, Portugal). Transfer buffer consisted of 0.025 mol L^−1^ Tris, pH 8.3, 0.192 mol L^−1^ glycine, and 200 mL L^−1^ methanol. Unless otherwise stated, chemicals were provided by Sigma-Aldrich (Merck KGaA, Darmstadt, Germany). Horseradish peroxidase (HRP)/anti-M13 monoclonal mouse antibody was purchased from GE Healthcare (GE Healthcare UK Ltd., Amersham, UK).

The human domain antibody library (dAb), M13 K07 helper phage and *Escherichia coli* TG1 strain (K12Δ (lac-proAB) supE thi hsdD5/F’ traD36 proA^+^B lacl^q^ lacZΔM15) were obtained from Source BioScience (Nottingham, UK). This is a single-domain antibody library based on a VH framework (V3-23/D47) that was developed by Daniel Christ at the MRC Laboratory of Molecular Biology (Cambridge, UK) [17]. Diversity was introduced into the antigen binding domains by polymerase chain reaction (PCR) mutagenesis into the three complementarity-determining regions (CDR1, CDR2 and CDR3). The library is constructed in the ampicillin resistant phagemid vector pR2 (MYC VSV tag) with a size of 3 × 10^9^. The repertoire has been engineered to withstand heat-induced aggregation on phage and has been displayed as a fusion with the terminal phage gene III protein.

### 2.2. Sample Preparations

A wide variety of tree nuts, plant and animal species (Table 1), commercial food products and commercial pistachios (raw and roasted) from different origins (the United States, Iran, and Spain) were analysed. The samples were acquired in different stores and local markets in Madrid (Spain). All food samples (5 g) were finely ground and stored in screw capped vials at −20 °C.

Pistachio, cashew, and peanut were defatted to be used in some of the biopanning procedures. The nut (5 g) was finely ground in an analytical mill (IKA^®^ A11, Staufen, Germany), and 0.8 g of nut powder was dissolved in 20 mL of acetone, vigorously shaken for 1 min, and centrifuged at 10,000× *g* for 30 min. Then, the supernatant was removed, and the process was repeated four times. Finally, the defatted sediment was dried for 24 h and stored in screw capped vials at −20 °C.

Binary mixtures (10^5^ to 100 mg kg^−1^) of roasted pistachio in corn flour were prepared using an IKA A11 analytical mill to evaluate the sensitivity of the assay and to be used as reference samples. Concentration of 10^5^ mg kg^−1^ was prepared by adding 5 g of ground pistachios to 45 g of corn flour. Concentrations of 10^4^, 10^3^, and 100 mg kg^-1^ were prepared by adding 5 g of the corresponding binary mixture to 45 g of corn flour. Concentrations of 7.5 × 10^4^, 5 × 10^4^, 2.5 × 10^4^, 2 × 10^4^, 5 × 10^3^, and 500 mg kg^−1^ were prepared in a similar way. All the mixtures were stored in screw capped vials at −20 °C.

Protein extracts from binary mixtures, tree nuts or food products were prepared by mixing 0.2 g sample with 1.2 mL of protein extraction buffer, and the mixture was shaken for 10 min at room temperature in a vertical rotator (HulaMixer Sample Mixer, Invitrogen, Carlsbad, CA, USA). The slurry was centrifuged at 10,000× *g* for 10 min at 4 °C, and the supernatant was filtered through a 0.45 µm syringe filter (Sartorius AG, Goettingen, Germany).

DNA extraction and purification for real time PCR analysis of samples was performed as previously described [11]. The DNA obtained from the Wizard DNA Clean-Up System kit (Promega, Madison, WI, USA) was eluted in 50 µL of sterile deionised water, and DNA concentration was measured with a NanoDrop ND-1000 spectrophotometer (NanoDrop Technologies Inc., Montchanin, Denmark).

A negative control, without sample, was included in every protein or DNA extraction. All protein and DNA extracts were stored at −20 °C.

### 2.3. Sequence Analysis

DNA from single colonies of the pistachio-recognizing clones was amplified using My Taq Mix 2× (Bioline Reagents Limited, London, UK) and primers CDR1-2 Fw (5′-ACGTCAGAAGACATCAGGTGCAGCTGTTGGAGTC-3′) and CDR3 Rev (5′-TCAGTTGAAGACCTCGAATTCAGATCCTCTTCTGAGATG-3′) [18]. The polymerase chain reaction (PCR) program used was 95 °C for 2 min, then, 95 °C for 15 s, 60 °C for 15 s, 72 °C for 10 s for 30 cycles, and a final extension at 72 °C for 7 min. PCR products (360–390 bp) were examined by electrophoresis on 1.5% agarose gel.

To confirm the suitable sequence of the selected clones with a complete VH fragment, the phagemid DNA was extracted by High Pure Plasmid Isolation kit (F. Hoffmann-La Roche Ltd., Basel, Switzerland) and sequenced with primers M13 Rev (5′-CAGGAAACAGCTATGACC-3′) and pR2seq (5′-CCCTCATAGTTAGCGTAACGA-3′) at Unidad de Genómica (Universidad Complutense de Madrid) with a multi-capillary sequencer “ABI Prism 3730” (Applied Biosystems, Waltham, Massachusetts, USA).

Nucleotide sequences were compared using European Molecular Biology Open Software Suite (Emboss software), and then analysed with IMGT/V-QUEST (sequence alignment software for IG and TR) to determine framework and complementary determining regions (CDR) of the VH. Amino acid sequences were deduced from the nucleotide sequences by SnapGene software (Insightful Science; available at snapgene.com).

### 2.4. Biopanning Procedure

The phage display dAb library was prepared as recommended by the manufacturer [17]. Following amplification of the library and precipitation of the phage particles, the pellet was resuspended in 1 mL of PBS and centrifuged at 11,600× *g* to remove any bacterial debris. The phages were titrered and kept at 4 °C for short-term storage (one week) or at −80 °C in 15% glycerol for long-term storage.

Different biopanning strategies (S1–S4) were carried out to obtain pistachio specific clones (Table 2). In all the strategies, polystyrene paddles were used as solid phase for the first round of biopanning and magnetic beads for the second round to avoid the isolation of nonspecific binders that would produce false-positive results. For the first round of selection, polystyrene Nunc paddles (Thermo Fisher Scientific, Waltham, MA, USA) with a surface area of 5.2 cm^2^ were coated with 1 mL of 100 μg mL^−1^ pistachio extract (positive screening) or peanut or cashew nut extract (negative screening) in PBS and incubated overnight at 4 °C. Then, paddles were washed three times with PBS and blocked with MPBS at 37 °C for 1 h. For the second round of selection, Dynabeads M-280 Tosylactivated (Thermo Fisher Scientific) were used to bind 100 µg of the target proteins following the manufacturer’s instructions.

The biopanning processes were performed as described in Madrid et al. [19], but using roasted pistachio, cashew, and peanut whole or defatted extracts, as shown in Table 2.

For the selection of pistachio-specific phage-dAb, two rounds of selection were carried out in each strategy. In the first round, approximately 5 × 10^12^ amplified phages from the dAb library were diluted in 1 mL of MPBS and added to the appropriate polystyrene paddles for a first negative selection. The mixture was incubated at 25 °C for 1 h to capture the phage dAbs that bind cashew or peanut, depending on the strategy. Then, the mixture containing the uncaptured phage-dAbs was added to the paddle coated with pistachio and incubated at 25 °C for 1 h with rotation and for another hour without rotation. Unbound phages were then removed by washing 10 times with PBS, and phages specifically bound to pistachio proteins were eluted by adding 1 mL of trypsin solution (1 g L^−1^ trypsin in PBS) for 1 h at room temperature with rotation. Ten microliters of eluted phage were used for titration, and 500 μL was used to infect 30 mL of *E. coli* TG1 culture at an OD_600_ of 0.5, and the flask was incubated for 1 h at 37 °C in a water bath. Infected cells (1 mL) were spread on six TYE agar plates containing 100 μg mL^−1^ ampicillin and 4% (*w*/*v*) glucose and grown overnight at 37 °C. Following overnight incubation, *E. coli* colonies were scraped into 2 mL of 2 × TY containing 15% glycerol and stored at −80 °C (labelled as first round stock). To amplify the phages for the second round of selection, 1 mL of recovered bacteria from the first panning (or until OD_600_ of 0.1) were inoculated into 500 mL of 2 × TY containing 100 μg mL^−1^ ampicillin and 4% (*w*/*v*) glucose and incubated at 37 °C until reaching an OD_600_ of 0.5. Then, infection with helper phage and precipitation was performed as described in Lee et al. [17]. A second round of selection was performed like the first one but employing 5 mg of Dynabeads instead of polystyrene paddles and increasing the number of washes to 20.

To select pistachio-binding clones, 95 individual colonies from the titration plates of the second round of selection were randomly picked and inoculated in separate wells of Nunc cell culture microplates (Thermo Fisher Scientific) containing 200 μL 2 × TY with 100 μg mL^−1^ ampicillin and 4% (*w*/*v*) glucose. Following infection with the helper phage, monoclonal phage-dAbs from the culture supernatant were screened for pistachio binding.

### 2.5. Indirect Phage-dAb Enzyme-Linked Immunosorbent Assay (ELISA)

Flat-bottom polystyrene microtiter plates (F96 MaxiSorp Nunc immunoplates (Thermo Fisher Scientific) were coated with the appropriate dilutions of the protein extracts assayed (pistachio, heterologous species, experimental mixtures, or commercial products) diluted 1:100 in PBS and incubated at 37 °C for 1 h. Then, the plates were washed five times and blocked with 200 μL of MPBS per well for 1h at 37 °C. After the plates were washed five times, culture supernatant (25 µL) in 100 μL of PBS with 3% (*w*/*v*) BSA or 100 μL of MPBS containing 2 μL of precipitated phage was added to each well, and the plates were incubated for 1 h at room temperature with shaking. After washing 10 times, plates were incubated at room temperature for 1 h with 100 μL of HRP/anti-M13 monoclonal mouse antibody (GE Healthcare UK Ltd., Amersham, UK) diluted 1:5000 in MPBS. Following another washing step, the reaction was developed with tetramethylbenzidine substrate solution (100 μL) for 12 min in the dark and stopped with 50 μL 1 M sulphuric acid. OD_450_ was measured with a spectrophotometer (FLUOstar Optima, BMG Labtech, Ortenberg, Germany). All washing steps were performed with PBS. All experiments were performed in triplicate. The experimental mixtures containing 5 × 10^3^, 10^4^, and 10^5^ mg kg^−1^ pistachio, and also negative control wells (for antigen, phage-dAb and secondary antibody), were analysed along with the food samples (heterologous species and commercial samples) as references and controls.

The concentration-response curve obtained by plotting the absorbance values vs. the log of pistachio protein concentration was fitted to the four-parameter logistic equation using Origin 8.0 software (OriginLab Corp., Northampton, MA, USA).

The limit of detection (LOD) was calculated as the concentration of the target protein that presents an absorbance value higher than the average of the eight non-target tree nuts plus three times its standard deviation (SD).

### 2.6. Protein Fractionation of the Pistachio Extract by Size-Exclusion Chromatography

Size-exclusion chromatography separation was carried out in a fast protein liquid chromatography system (ÄKTA purifier FPLC system) (GE Healthcare UK Ltd., Amersham, UK). One hundred microliter of defatted and filtered pistachio protein extract was injected into a HiPrep 16/60 Sephacryl S-200 HR column (GE Healthcare UK Ltd., Amersham, UK) previously equilibrated with PBS. The flow rate was maintained at 0.5 mL min^−1^. Eluted fractions were collected in 1.5 mL glass vials and stored at −20 °C until further use.

### 2.7. SDS-PAGE and Western Blotting Analysis

Duplicate sodium dodecyl sulphate polyacrylamide gel electrophoresis (SDS-PAGE) of the size exclusion chromatography fractions (5 µg/lane) and pistachio extracts (10 µg) were performed using precast gels (4–20% TDX, Bio-Rad, Hercules, CA, USA) in a Mini-Protean Tetra Cell (Bio-Rad) at 120 V for 45 min. Each sample was mixed with 10 µL of sample buffer and heated at 95 °C for 5 min before addition to the wells. Following electrophoresis, one of the gels was stained with Blue safe (NZytech, Lisbon, Portugal), and protein bands in the other gel were transferred into a methanol-activated polyvinylidene difluoride (PVDF) membrane (Immuno-Blot PVDF membranes; Bio-Rad) at 240 mA for 2 h using a Mini Trans-Blot Cell (Bio-Rad). The membrane was then blocked with 3% BSA in PBS for 1h at 25 °C, washed three times with PBS, and incubated overnight at 4 °C with pistachio specific phage-dAb (750 µL of PVF4 clone culture supernatant diluted with 2.25 mL of 1% of BSA in PBS). After washing five times with PBS, the membrane was incubated for 1 h at 37 °C with HRP/anti-M13 mouse monoclonal antibody (GE Healthcare UK Ltd., Amersham, UK) diluted 1:5000 in 1% BSA-PBS, washed three times with PBST and revealed with the chemiluminescent substrate Clarity Western ECL (Bio-Rad). The Western blotting membranes were scanned using a ChemiDoc XRS system (Bio-Rad) to visualise bands.

### 2.8. Protein Identification

Bands of interest from the Blue Safe stained SDS-PAGE gel were cut out with a sterile scalpel and immersed in 5% acetic acid solution. Proteins were in gel reduced, alkylated, and digested with trypsin according to Sechi and Chait [20]. Analysis of peptides from protein digestion was performed using the 4800 Plus MALDI-TOF/TOF (matrix-assisted laser desorption/ionization tandem mass spectrometry) Analyzer mass spectrometer (Applied Biosystems, MDS Sciex, Toronto, ON, Canada), at the Unidad de Proteómica of Universidad Complutense de Madrid (Spain). Peptide mass fingerprint and some peptide fragmentation spectra were combined searched in the MASCOT v2.3 search engine (http://www.matrixscience.com) through Global protein Server (Applied Biosystems) against NCBI database (17,919,084 sequences; 6,150,218,869 residues) without taxonomy restriction and search parameters: carbamidomethylcysteine as fixed modification and oxidised methionine as variable modification; peptide mass tolerance 80 ppm; one missed trypsin cleavage site allowed and MS/MS fragments tolerance 0.3 Da. In all protein identification, the probability scores were greater than the score fixed by Mascot as significant with a *p*-value minor than 0.05.

## 3. Results and Discussion

### 3.1. Screening of the Domain Antibody (dAb) Library in Search for Pistachio-Specific Clones

Domain antibody (dAb) libraries are novel tools in the phage display technology for the obtention of recombinant antibody fragments. Compared with Fab or scFv libraries [21], randomization in dAb constructions can be introduced at a much higher percentage of CDR positions without exceeding practical library size, being simpler and more efficient.

Pistachio and cashew belong to the *Anacardiaceae* family, and due to its phylogenetic proximity, it is difficult to obtain pistachio-specific antibodies that do not have a cross reaction with cashew [2]. A commercial dAb library has been searched in a phage display format to obtain specific clones for detection of pistachio in processed foods. Several biopanning strategies (S1–S4) involving two rounds of selection were carried out using protein extracts from whole (S1 and S2) or defatted (S3 and S4) pistachio, cashew, and peanut (Table 2).

**Figure 1:**
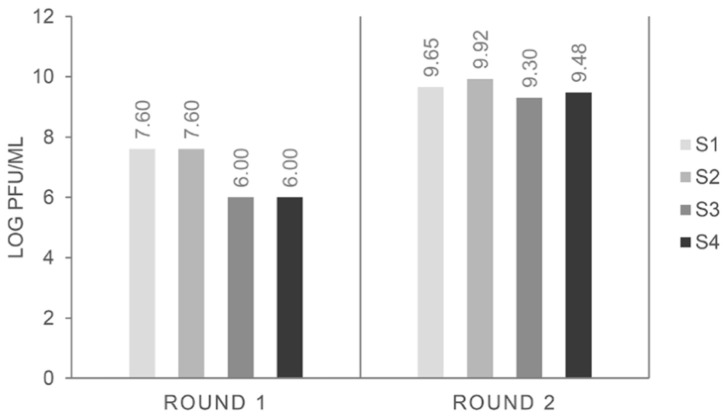
Phage titers obtained after each round of affinity selection against pistachio extract, following different biopanning strategies (S1–S4).

The input number of phage particles was always around 5 × 10^12^ pfu mL^−1^ and, as expected, the phage particles recovered at the end of the first round were around 10^5^–10^7^ pfu mL^−1^, increasing up to 10^7^–10^9^ in subsequent rounds of selection [17]. That was the case for all biopanning strategies (Figure 1), and it could be indicative of an increase in the selection of binders to pistachio.

**Figure 2:**
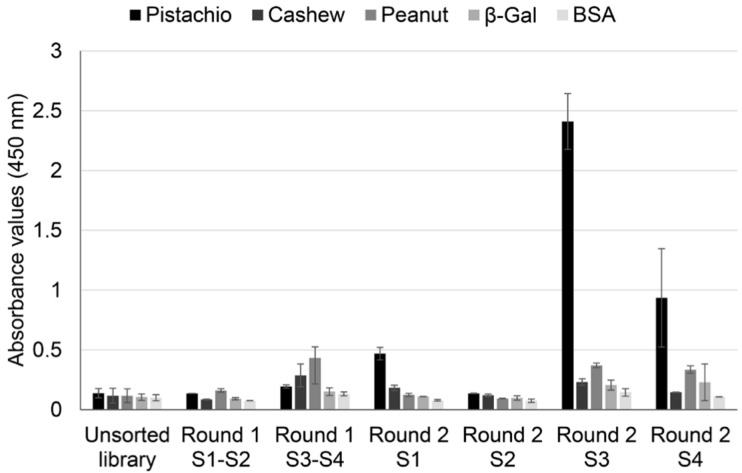
Indirect phage-dAb ELISA results obtained with polyclonal phages rescued at each round of selection from strategies S1–S4 against pistachio. Precipitated phages from each round of selection were analysed against pistachio, cashew and peanut extracts and negative controls (beta-galactosidase and BSA). Absorbance values are the mean of three independent experiments with duplicates. Error bars show the standard deviation for each set of data.

To confirm this hypothesis, a polyclonal phage ELISA against pistachio, cashew, and peanut was performed with phage pools collected from all the rounds of selection, using BSA and β-galactosidase as negative controls (Figure 2).

The results showed an increase in absorbance values for pistachio proteins after the second rounds, with important differences between strategies. When whole protein extracts were used as a target, pistachio binding increased only slightly in S1, and it did not increase further in S2. On the contrary, using defatted extracts as target allowed a clear enrichment in pistachio binding phage-dAb, with higher values registered in S3 (subtraction of peanut-binding phages in rounds 1 and 2 before selection of pistachio-binding phages) than in S4 (subtraction of peanut-binding phages in round 1 and cashew-binding phages in round 2). This result could mean that a negative selection against cashew produced recovery of a lower number of pistachio-binding phages but more specific to the pistachio target. Very low cross-reactivity was found to wells coated with BSA, β-galactosidase, cashew, and peanut. Thus, according to these results, the use of defatted extracts increased the efficiency of the biopanning process, so that the phage population that specifically recognised pistachio was highly enriched in the second round of panning, avoiding the need for additional rounds of selection.

### 3.2. Screening of Individual Phage-dAb Clones by Monoclonal Phage ELISA

Monoclonal phage ELISA was performed to isolate and identify clones producing dAb that recognised pistachio proteins. Ninety-five *E.* coli TG1 colonies from the titration plates of the second rounds of panning of the different strategies were picked, and phage-dAbs produced in the culture supernatant were analysed. The clones were considered positive when binding to pistachio extract was at least five times higher than binding to the peanut or cashew extracts used as negative controls (A_450_ against pistachio > 5 × A_450_ against negative control).

When the negative selection was made with peanut (S1), two clones recognizing pistachio were obtained (PVD5 and PVF10), but they also cross-reacted with cashew (Table 2). It is possible that negative subtraction with peanut eliminated clones recognizing other tree nuts, but the remaining pistachio-binding clones also recognised the phylogenetically close cashew. In strategy S2, the negative selection was performed with peanut in round 1 and with cashew in round 2. This strategy yielded just one positive clone that exclusively recognised pistachio (PVG3). Finally, this clone was discarded because its nucleotide sequence revealed that it did not codify for a complete dAb, generating a truncated antibody without the signal factor, the FR1, and part of the CDR1.

**Figure 3:**
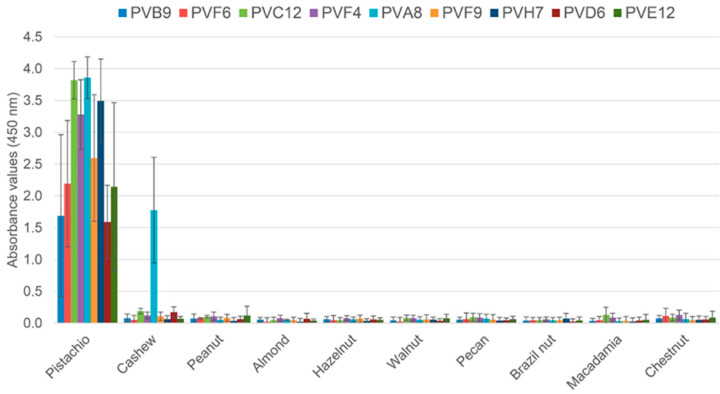
Indirect phage-dAb ELISA results obtained with monoclonal phages selected from the second round of biopanning against a set of tree nuts. Absorbance values are the mean of four independent experiments with duplicates. Error bars show the standard deviation for each set of data.

Pistachios are characterised by a high fat content (between 40.6–53.5% g per 100 g) with a heart-healthy fatty acid profile [22,23]. They are also rich in polyphenols (around 600 mg per 100 g) [24]. Polyphenols are known to form complexes with proteins leading to changes in the structural, functional and nutritional properties of both compounds [25]. Considering that the high fat content of pistachio and other tree nuts could be hindering obtention of pistachio specific dAb, the same biopanning strategies used with whole extracts (S1 and S2) were performed but using defatted extracts of pistachio, peanut and cashew. In the strategy S3 a total of 13 pistachio binding clones were obtained out of the 95 clones analysed (13.7%) from the second round (Table 2). Three of them showed cross reaction with the cashew extract (PVA7, PVA8 and PVD8), and the remaining clones bound only pistachio (PVB4, PVB9, PVC11, PVC12, PVD6, PVE12, PVF6, PVF9, PVG8, and PVH7). In the S4 strategy, there were four pistachio-binding clones out of 95 clones analysed from the second round (4.2%). Three of them were considered specific to pistachio (PVA1, PVA12, and PVF4), and only one cross-reacted with cashew (PVB12). Following initial screening, eight clones from the second rounds of S3 and S4 were selected for further analysis in a monoclonal ELISA with phage-dAb to assess specificity against ten allergenic nut species (Figure 3). The eight selected clones bound pistachio with ABS_450_ values higher than 1.5, without cross-reactivity to the rest of the nuts. Only the clone PVA8 (S3) cross-reacted to the cashew extract, and it was then discarded.

These results demonstrated that negative biopanning with a closely related but non-target protein (cashew extract) excluded a part of the pistachio reactive phages, but still allowed selection of the most specific phage-dAb. Moreover, the use of defatted extracts was necessary to obtain pistachio-specific clones. The defatting protocol (Section 2.1) was performed with acetone, which is a good method to eliminate fatty acids and also improves the extraction of polyphenols [24,26]. Therefore, the extracts used in strategies S3 and S4 are characterised by a high protein content without elements such as fatty acids and phenols that interact or prevent recognition of the protein epitopes by dAb, thus improving the efficiency of the selection of phage antibodies.

### 3.3. Sequence Analysis

**Figure 4:**
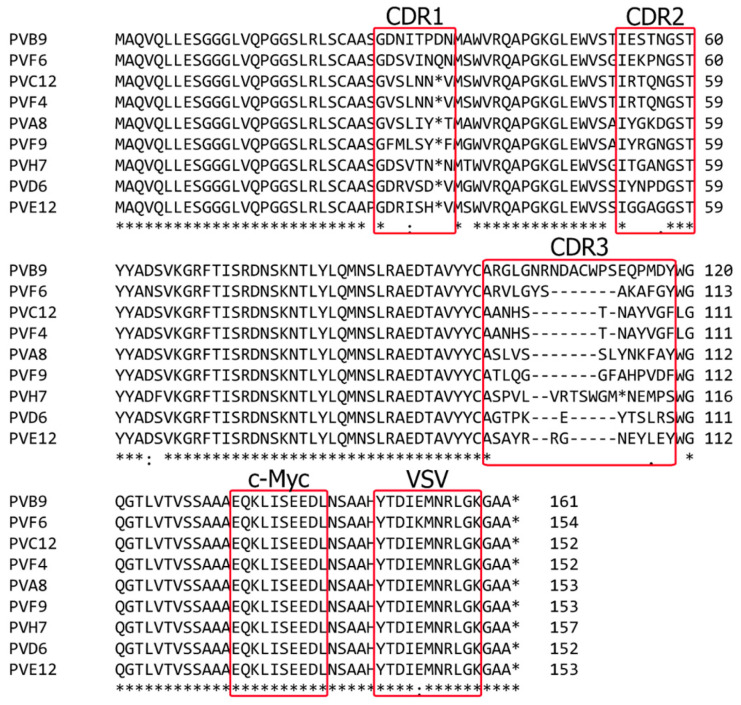
Amino acid sequences of the pistachio binding dAbs deduced from the nucleotide sequences by SnapGene tool. Positions of the complementarity determining regions of the variable domains (CDR 1–3) and the c-Myc and VSV tags are indicated as determined by IMGT/V-QUEST tool.

DNA from the eight pistachio-specific clones selected, and one of the clones cross-reacting with cashew, was amplified by PCR with primers CDR1-2 and CDR3 to estimate the proportion of clones containing the complete VH insert (approximately 360–390 bp); all of them produced PCR fragments of the expected size (not shown). The inserts of the purified plasmids were then sequenced, and the sequences were uploaded to GenBank with the following accession numbers: PVA8-dAb (MN862009), PVB9-dAb (MN631054), PVD6-dAb (MN862010), PVE12-dAb (MN862011), PVF4-dAb (MN612110), PVF6-dAb (MN631055), PVF9-dAb (MN862012), and PVH7-dAb (MN862013). When compared to the NCBI database, the selected positive clones were confirmed to be *Homo sapiens* partial IGHV3-23 gene for immunoglobulin heavy chain variable region. Finally, DNA sequence analysis of the nine clones and their deduced amino acid sequences (Figure 4) demonstrated that they were all different except dAbs PVF4, obtained with strategy S4, and PVC12, obtained with strategy S3, that had the same nucleotide sequence. The clone PVF4 was then selected because of its higher culture stability and phage-dAb production.

Deduced amino acid sequences of the pistachio-binding clones showing the CDRs and immunoglobulin framework regions (FRs) demonstrated no insertions or deletions in CDR1 and CDR2 in any of the clones. As expected, CDR3 was the most variable in sequence and length among the selected clones, with lengths from 12 to 20 residues. Moreover, a stop codon was present in the CDR1 of six clones, and in CDR3 or one clone, due to an amber mutation (Figure 4). This is frequent in phage displayed antibodies because of randomization of CDR sequences and propagation of phagemids in the amber suppressor TG1 *E. coli* strain [27]. Amber stop codons are interpreted as Gln residues in suppressor strains like TG1, so that these dAb clones can be used in a phage display format, but require site directed mutagenesis to be produced as soluble dAbs in a non-suppressor strain.

The target-binding site of human antibodies is usually composed by the variable domains of the heavy and light chains (VH and VL), that are non-covalently associated, and together contribute for proper target binding. However, there are naturally occurring and genetically engineered single chain antibodies that maintain their ability to recognise antigens with high affinity and specificity [15,17]. That is the case of the human-domain antibody library (dAb) used in this work, which consisted of antibody fragments based exclusively on a single VH domain [17]. Advantageous features of dAbs include their small size (15 kDa), high solubility and stability, and excellent tissue penetration in vivo. Domain antibodies can be genetically linked to peptide tags as c-Myc or His tags to facilitate their purification when produced as soluble antibody fragments.

### 3.4. Identification of Pistachio Proteins Recognised by the PVF4 Phage-dAb

To identify the protein fractions recognised by the PVF4 phage-dAb, a chromatographic separation of the defatted pistachio extract was performed by FPLC, followed by an indirect phage-dAb ELISA and a Western blot with FPLC fractions.

**Figure 5:**
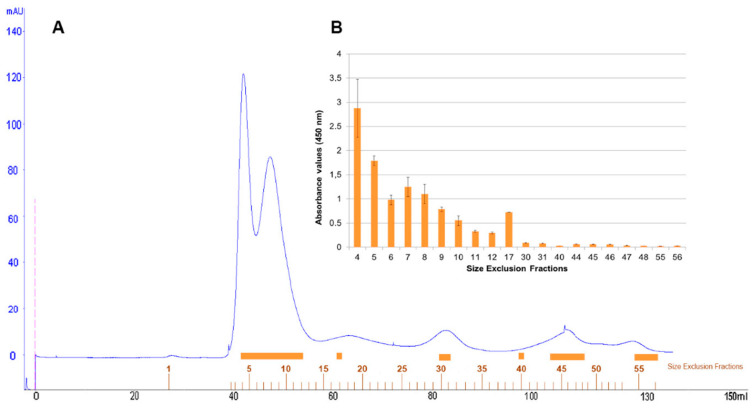
Elution profile of pistachio proteins obtained from preparative FPLC size exclusion column (**A**) showing absorbance values at 280 nm. (**B**) Indirect phage-dAb ELISA with some fractions obtained from FPLC (indicated by an orange bar). Absorbance values are the mean of duplicates. Error bars show the standard deviation for each set of data.

Size-exclusion chromatography with FPLC system of defatted pistachio protein extract was resolved in a protein profile with two major peaks and three minor peaks at 280 nm (Figure 5A). One hundred microlitres of representative protein fractions obtained from size-exclusion chromatography were analysed in an indirect-phage ELISA using PVF4 phage-dAb (Figure 5B).

**Figure 6:**
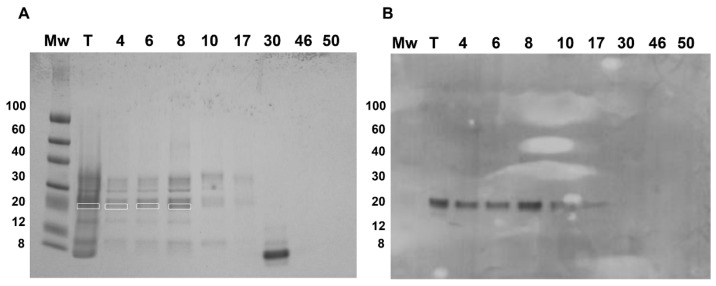
Sodium dodecyl sulphate polyacrylamide gel electrophoresis (SDS-PAGE) in reducing conditions of different fractions obtained in size exclusion chromatography (**A**) and immunoblot with PVF4 phage-dAb (**B**) of 10 µg of pistachio extract (T) and 10 µL of each fraction sample. Mw of the protein marker bands (ColorBurst^TM^ Electrophoresis Protein Marker, Merck KGaA, Darmstadt, Germany) is indicated. Highlighted bands were excised and analysed by matrix-assisted laser desorption/ionization tandem mass spectrometry (MALDI-TOF/TOF). Phage-dAb in the immunoblot was detected with anti-M13 monoclonal mouse antibody conjugated with Horseradish peroxidase (HRP).

Fractions 4–17, belonging to the two major and first minor peaks, were detected by the PVF4 phage-dAb. In order to identify the specific proteins recognised by PVF4 phage-dAb, an SDS-PAGE of the pistachio extract and selected fractions was performed under reducing conditions. The most prominent electrophoretic bands had molecular weights ranging from ∼20 kDa to ∼35 kDa (Figure 6A).

The electrophoretic and Western blot patterns of total pistachio extract and fractions 4–17 were similar to those reported by Liu et al. [10] under reducing conditions with a murine monoclonal antibody against pistachio. A unique band of ∼22 kDa was detected in the Western blot by the PVF4 phage-dAb (Figure 6B). According to Liu et al. [10], the target antigen is likely to be the basic subunit of the pistachio 11S globulin (allergen Pis v 2) that belongs to the cupin superfamily of proteins, and has been identified as a major pistachio allergen [4]. To verify the hypothesis, the bands from total extract and fractions 4, 6, and 8 recognised by the PVF4 phage-dAb were excised and analysed by matrix-assisted laser desorption/ionization tandem mass spectrometry (MALDI-TOF/TOF). As shown in Table S1, the peptides obtained from all the bands were identified in the amino acid sequence of the 11S globulin (*Pistacia vera*) (accession number: ABU42022). The coverage of the 11S sequence obtained was 45% in the case of lane T, 22% for lane 4, 30% for lane 6, and 25% for lane 8 (Table S1).

**Figure 7:**
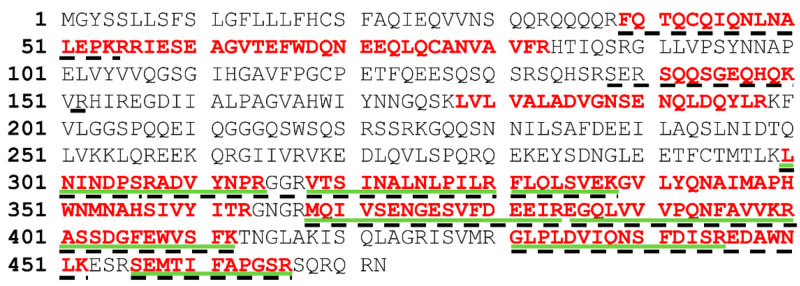
Amino acid sequence of the 11S globulin (*Pistacia vera*) (accession number: ABU42022). Positions of peptides identified by matrix-assisted laser desorption/ionization tandem mass spectrometry (MALDI-TOF/TOF) from lane ‘T’ in the Western blot are in red bold font. Positions of the peptides identified from the lane ‘4’ are underlined with green solid lines. Positions of the peptides identified from lanes ‘6’ and ‘8’ are discontinuous underlined.

Here, 11S globulins are defined as bicupins owing to the presence of two cupin domains, each presenting a beta-barrel motif. They are non-glycosylated multimeric structures (hexamers or mixture of trimers) synthesised as a single polypeptide, which is post-translationally cleaved into an acidic (30–40 kDa) and a basic (22–27 kDa) polypeptides linked by a disulphide bond [4,5,10]. It should be noted that most of the peptides obtained from lanes 4, 6, and 8, aligned to the C-terminal region of the protein sequence, while peptides in the band from the total extract aligned throughout the whole sequence (Figure 7).

**Figure 8:**
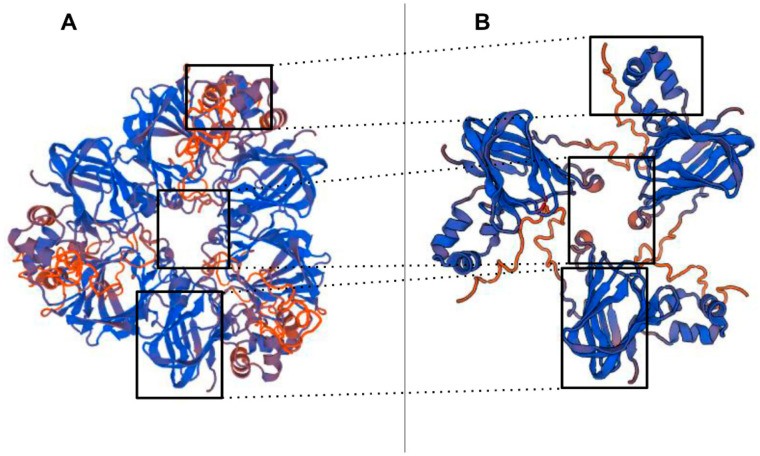
The 11S globulins are hexameric proteins with subunits comprising an acidic polypeptide 30–40 kDa in size that is disulphide-linked to a 20 kDa basic polypeptide. (**A**) SWISS-MODEL of a homo-trimer of pistachio 11S globulin seed storage protein (ABU42022), (**B**) SWISS-MODEL of the basic subunit homo-trimer recognised by PVF4-dAb, the rectangles show the possible epitope recognition zones.

The 3D structure of the pistachio 11S globulin (ABU42022) identified as target of the PVF4 phage-dAb by MALDI-TOF/TOF, was predicted by homology modelling, using the SWISS-MODEL server (Figure 8) https://swissmodel.expasy.org/ [28]. The model was built through comparison to the closely related sequence and structure of the 11S globulin seed storage protein from *Amaranthus hypochondriacus* L., 3qac.1.A, which served as template. Most of the peptides identified by MALDI-TOF/TOF, seem to belong to the basic protein subunit (Cupine-1) of the 11S globulin [2,4]. This result agrees with Liu et al., [10], indicating that pistachio 11S globulin basic subunit is a good antigen for producing antibodies targeting human allergy relevant epitopes.

### 3.5. Assay Specificity and Detection Limit

Indirect phage ELISA assay using precipitated phage-dAb antibody from clone PVF4 was able to detect pistachio proteins from all the raw and roasted pistachio kernels of different origins that were analysed (Spain, Iran, and the US). Roasted pistachios were used for the biopanning process in search for pistachio-specific recombinant antibodies because heat treatments may affect the solubility and integrity of the nut proteins and limit their recognition in the ELISA [29].

**Figure 9:**
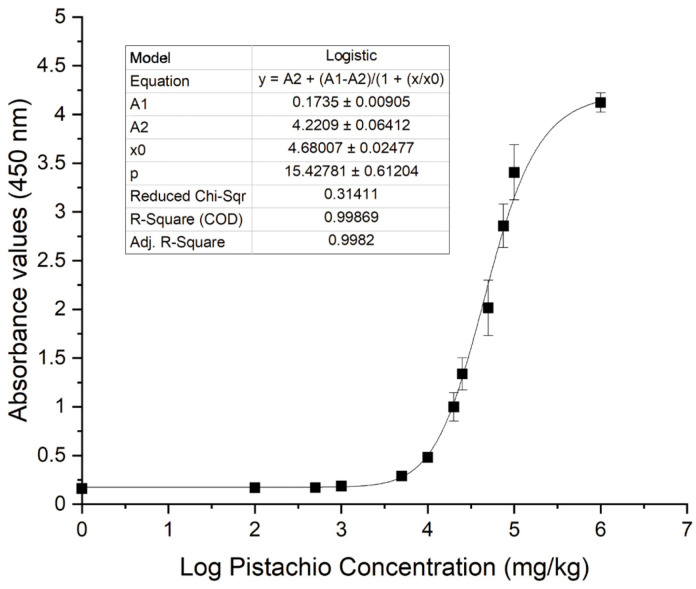
Standard curve of the phage-dAb enzyme-linked immunosorbent assay (ELISA) performed with protein extracts obtained from ground defatted pistachio (■) in corn flour binary mixtures. The plot shows the average values and the standard deviations corresponding to duplicate experiments performed in three different days.

Experimental pistachio/corn flour mixtures instead of pistachio extract dilutions were prepared and analysed in the indirect phage-dAb ELISA to assess sensitivity because the food matrix may affect assay performance due to the presence of interfering matrix components. It was demonstrated that the assay was able to detect pistachio proteins in a corn flour matrix in a concentration-dependent manner (Figure 9). The four-parameter logistic equation obtained with the experimental data was *y* = A_2_ + (A_1_ − A_2_)/(1 + (x/x_0_)^p); being A_1_ = 0.1735, A_2_ = 4.2209, x_0_ = 4.68007, *p* = 15.42781 and *R^2^* = 0.99869. The limit of detection (LOD) of this indirect phage-dAb ELISA was 3983 mg kg^−1^. The coefficients of variation were in the range of 2–15% for mixtures containing pistachio above the LOD. This LOD is higher than the detection limits reported for other immunoassays and commercial kits available to detect pistachio using polyclonal or monoclonal antibodies. The LOD of pistachio nut protein reported for the MonoTrace pistachio ELISA kit (BioFront Technologies, Tallahassee, FL, USA) and AgraQuant ELISA pistachio (Romer Labs, Tulln, Austria) are 0.12–0.13 mg kg^−1^ in various matrices, while the LOD for several lateral flow devices (LFD) are in the range of 1–10 mg kg^−1^ [5,10].

Cross-reactivity of the assay was calculated as the amount of pistachio estimated by interpolating the absorbance values of the heterologous species in the logistic equation. Cross-reactivity assays using PVF4 phage-dAb showed that none of a wide variety of plant and animal species (Table 1) developed an absorbance signal higher than the LOD of pistachio proteins. Only the egg yolk extract produced a cross-reaction of 4.30%, but not the egg white. Even though this could produce some false positives in products containing egg yolk, most commercial products declaring egg usually contain egg white, which also contains most of the egg allergens [30] but does not cross-react in this assay. However, in contrast to other published immunoassays, that present a high cross-reaction with cashew (approximately 12% [5], or even higher than 90% [31]), the PVF4 phage-dAb does not cross-react with cashew. Hence, taken together these results suggest that the PVF4 phage-dAb ELISA is specific but not as sensitive as the previously developed real time PCR technique [11] for pistachio (0.1 mg kg^−1^), or other published ELISA methods using polyclonal or monoclonal antibodies [32].

### 3.6. Analysis of Commercial Food Products

The applicability of the indirect phage ELISA method developed for detection of pistachio proteins in foodstuffs was assessed through analysis of 77 commercial products acquired at different stores of Madrid, including bakery and pastry products, energy bars, chocolates, ice creams, yoghurts, beverages, sauces and prepared dishes. The samples were classified into four groups depending on their content in pistachio and other nuts as declared in their labelling (Table 3).

Among the samples analysed, 34 were labelled as containing pistachio as ingredient, five labelled as ‘pistachio traces’, 29 declared to contain other tree nuts different than pistachio or tree nut traces, and the remaining nine did not declare to contain any nuts or traces thereof. In order to confirm the results obtained in the phage ELISA, samples were also analysed by the pistachio-specific real-time PCR method previously developed in our laboratory [11].

From 34 commercial products containing pistachio in the list of ingredients, only a chocolate sample tested negative by real-time PCR. Nevertheless, 14 samples tested negative for pistachio by phage-dAb ELISA, including biscuits (2), cake (1), cold meat (5), chocolate (2), pate (1), breakfast cereal (1), ice-cream (1), and snack (1). Negative ELISA results could be explained either because the pistachio concentration in the products is below the LOD or because of a denaturation of the epitope recognised by the phage-dAb due to food processing. In the case of the biscuits, chocolate, and cake samples that tested negative, the pistachio content was not specified in the label, and it could be below the LOD. Some samples that tested negative by ELISA but positive by PCR declared their pistachio content, like the cold meats (0.2–1%), pate (35% boiled pistachio), ice-cream (1.5%), and snack (5%), but they are heat treated products, where the processing temperature may have produced the unfolding of the tertiary structure of the proteins and the aggregation of protein chains, avoiding epitope binding by the phage-dAb [29,33].

Among the five products that included precautionary advisory labelling regarding pistachio, one was positive only by PCR (a cold meat), and two samples tested positive by both methods (a stuffed pasta and a sausage).

In the group of 29 samples with labels indicating that they contain tree nuts other than pistachio or tree nut traces, all samples were negative by the phage-ELISA and real-time PCR methods, verifying a correct food labelling. It should be noted that 10 of the samples declared cashew nuts in the ingredient list and all tested negative in both ELISA and PCR pistachio methods, demonstrating the absence of cross-reaction with cashew. Moreover, those samples tested positive in a cashew-specific real-time PCR method [34]. In the last group of nine nut-free products, all the samples were negative for pistachio.

## 4. Conclusions

To the best of our knowledge, this is the first time a pistachio-specific recombinant antibody has been produced for detection of allergenic pistachio in food products and the first immunoassay for this purpose that is produced without animal immunization. It was demonstrated that the best biopanning strategies to recover pistachio-specific phage-dAbs were those using defatted pistachio protein extract as the target. The LOD of the pistachio assay developed is higher than that of other reported immunoassays [5,10,31]. The PVF4-dAb targets the pistachio basic subunit of the 11S globulin, and it is absent from cross reactions with the closely related cashew nuts, that are common in commercial ELISAs for pistachio detection.

## Figures and Tables

**Table 1:** List of heterologous species analysed in the indirect phage enzyme-linked immunosorbent assay (ELISA).

Nuts		
Almond (*Prunus dulcis*)	Hazelnut (*Corylus avellana*)	Pecan nut (*Carya illinoinensis*)
Brazil nut (*Bertholletia excels*)	Macadamia (*Macadamia integrifolia*)	Pine nut (*Pinus pinea*)
Cashew nut (*Anacardium occidentale*)	Peanut (*Arachis hypogaea*)	Walnut (*Juglans regia*)
Chesnut (*Castanea sativa*)		
**Vegetal Species**		
Anise *(Pimpinella anisum*)	Garlic (*Allium sativum*)	Pinto bean (*Phaseolus coccineus*)
Apple (*Malus domestica*)	Green peppers (*Capsicum annuum*)	Plum (*Prunus domestica*)
Apricot *(Prunus armeniaca*)	Kiwifruit (*Actinidia deliciosa*)	Pomegranate (*Punica granatum*)
Asparagus (*Asparagus officinalis*)	Lentil (*Lens culinaris*)	Poppy seed (*Papaver rhoeas*)
Aubergine (*Solanum melongena*)	Lupin bean (*Lupinus albus*)	Prune (*Prunus domestica*)
Banana *(Musa acuminate)*	Maize (*Zea mays*)	Pumpkin seed (*Cucurbita maxima*)
Barley *(Hordeum vulgare*)	Mandarin orange (*Citrus reticulate*)	Quinoa (*Chenopodium quinoa*)
Bean (*Phaseolus vulgaris*)	Melon (*Cucumis melo*)	Rice (*Oryza sativa*)
Blackberry (*Rubus ulmifolius*)	Oats (*Avena sativa*)	Rye (*Secale cereal*)
Brown sugar (*Saccharum officinarum*)	Olive (*Olea europaea*)	Sesame (*Sesamum indicum*)
Carrot (*Daucus carota*)	Onion (*Allium cepa*)	Soya (*Glycine max*)
Cherry (*Prunus avium*)	Orange (*Citrus sinensis*)	Sunflower seed (*Helianthus annuus*)
Chia (*Salvia hispánica*)	Paprika (*Capsicum annuum*)	Tiger nut (*Cyperus esculentus)*
Chickpea (*Cicer arietinum*)	Pea (*Pisum sativum*)	Tomato (*Solanum lycopersicum*)
Cinnamon (*Cinnamomum verum*)	Peach (*Prunus persica*)	Vanilla (*Vanilla planifolia*)
Cocoa (*Theobroma cacao*)	Pear (*Pyrus communis L.*)	Wheat (*Triticum aestivum*)
Flaxseed (*Linum usitatissimum*)	Pineapple (*Ananas comosus*)	Courgette (*Cucurbita pepo*)
**Animal Species**		
Beef (*Bos Taurus*)	Salmon (*Salmo salar*)	Chicken (*Gallus gallus domesticus*)
Egg (*Gallus gallus domesticus*)	Milk (*Bos Taurus*)	Pork (*Sus scrofa domestica*)

**Table 2:** Different biopanning strategies used, and number of specific pistachio-binding clones obtained from each strategy.

Strategies	Defatted Extracts	Selection ^a^	Round 1			Round 2			Clones Obtained
			Peanut	Cashew	Pistachio	Peanut	Cashew	Pistachio	
**S1**	NO	-	●			●			2
		+			●			●	
**S2**	NO	-	●				●		1
		+			●			●	
**S3**	YES	-	●			●			13
		+			●			●	
**S4**	YES	-	●				●		4
		+			●			●	

^a^ For each strategy and round of biopanning, a negative panning was first performed with the extract indicated by (−) to reduce non-specific binders. Then, the remaining phage-dAbs were used for selection of pistachio-specific phage-dAbs against the pistachio extract (+).

**Table 3:** Determination of the presence of pistachio in commercial processed food products using PVF4 phage-dAb ELISA and pistachio-specific real-time polymerase chain reaction (PCR).

Label Statement	Product	Number of Samples	Pistachio Phage-dAb ELISA ^a^	Real Time PCR ^a^
**Pistachio declared as ingredient**	Biscuit Cake Chocolate Cold Meat Pistachio Pate Breakfast Cereals Ice-cream Snack	2 5 3 14 1 1 2 6	−(2) +(4)/−(1) +(1)/−(2) +(9)/−(5) −(1) −(1) +(1)/−(1) +(5)/−(1)	+(2) +(5) +(2)/−(1) +(14) +(1) +(1) +(2) +(6)
**Pistachio Traces**	Biscuits Chocolate Cold Meat Stuffed Pasta Sausages	1 1 1 1 1	−(1) −(1) −(1) +(1) +(1)	−(1) −(1) +(1) +(1) +(1)
**Contain other tree nuts or may contain tree nuts traces**	Beverage Biscuit Bread Butter Cake Breakfast Cereals Cream Food Bar Ice-Cream Sandwich Sauce Snack Turron Yogurt	1 4 2 1 1 4 1 7 2 1 2 1 1 1	−(1) −(4) −(2) −(1) −(1) −(4) −(1) −(7) −(2) −(1) −(2) −(1) −(1) −(1)	−(1) −(4) −(2) −(1) −(1) −(4) −(1) −(7) −(2) −(1) −(2) −(1) −(1) −(1)
**Not declaring to contain nuts or traces**	Beverage Biscuit Bread Cake Breakfast Cereals Ice-Cream Snack	1 2 2 1 1 1 1	−(1) −(2) −(2) −(1) −(1) −(1) −(1)	−(1) −(2) −(2) −(1) −(1) −(1) −(1)

**^a^** Commercial food products showing estimated pistachio concentration lower than the limit of detection (LOD) in the phage-dAb ELISA, or Cp > 30 (equivalent to 1 mg kg^−1^ in PCR) were considered negative (−). The number of negative (−) and positive (+) samples detected are indicated in parenthesis.

## Supplementary Materials

The following is available online at https://www.mdpi.com/2304-8158/9/9/1230/s1, Table S1: Peptides identified by MALDI-TOF/TOF from electrophoretic bands recognised by the PVF4 phage-dAb in the pistachio extract (T) and in the chromatographic fractions 4, 6 and 8.
