# Supplementary material for: Phage Displayed Domain Antibodies (dAb) for Detection of Allergenic Pistachio Proteins in Foods
Source: Foods. 2020 Sep 3;9(9):1230. doi: 10.3390/foods9091230 (PMC7554873; doi:10.3390/foods9091230)
Supplement: Supplementary file 1 [file foods-09-01230-s001.pdf]

Article

Phage displayed domain antibodies (dAb) for detection of allergenic pistachio proteins in foods

Raquel Madrid[1], Aina García-García[1], Isabel González[1], Rosario Martín[1] and Teresa García[1*]

Supplementary material

Table S1. Peptides identified by MALDI-TOF/TOF from electrophoretic bands recognised by the PVF4 phage-dAb in the pistachio extract (T) and in the chromatographic fractions 4, 6 and 8.

Lane	Protein identification	Accession number	Sequence coverage	Total score	Ion scores	Peptide sequences
T	11S globulin [*Pistacia vera*]	gi|156001070	45 %	179		R.FQTQCQIQNLNALEPKR.R
						R.RIESEAGVTEFWDQNEEQLQCANVAVFR.H
						R.SQQSGEQHQK.V
						K.LVLVALADVGNSENQLDQYLR.K
						K.LNINDPSRADVYNPR.G
						R.VTSINALNLPILR.F
						R.FLQLSVEK.G
						K.GVLYQNAIMAPHWNMNAHSIVYITR.G
						R.MQIVSENGESVFDEEIR.E
						R.EGQLVVVPQNFAVVKR.A
						K.RASSDGFEWVSFK.T
						R.GLPLDVIQNSFDISREDAWNLK.E
						R.SEMTIFAPGSR.S
4	11S globulin [*Pistacia vera*]	gi|156001070	22 %	217		K.LNINDPSRADVYNPR.G
						R.VTSINALNLPILR.F
						R.FLQLSVEK.G
						R.MQIVSENGESVFDEEIR.E
					89	R.EGQLVVVPQNFAVVKR.A
						K.RASSDGFEWVSFK.T
						R.GLPLDVIQNSFDISR.E
					73	R.SEMTIFAPGSR.S
6	11S globulin [*Pistacia vera*]	gi|156001070	30 %	130		K.LNINDPSR.A
						R.FLQLSVEK.G
						R.ADVYNPRGGR.V
						R.SQQSGEQHQK.V
						R.SEMTIFAPGSR.S
						R.ASSDGFEWVSFK.T
						R.SQQSGEQHQKVR.H
						R.VTSINALNLPILR.F
						R.EGQLVVVPQNFAVVK.R
						R.GLPLDVIQNSFDISR.E
						K.LNINDPSRADVYNPR.G
						R.EGQLVVVPQNFAVVKR.A
						R.FQTQCQIQNLNALEPK.R
						R.MQIVSENGESVFDEEIR.E
						R.FQTQCQIQNLNALEPKR.R
						R.GLPLDVIQNSFDISREDAWNLK.E
8	11S globulin [*Pistacia vera*]	gi|156001070	25 %	276		R.SERSQQSGEQHQK.V
					47	K.LNINDPSR.A
					64	K.LNINDPSRADVYNPR.G
					114	R.VTSINALNLPILR.F
						R.FLQLSVEK.G
						R.MQIVSENGESVFDEEIR.E
						R.EGQLVVVPQNFAVVKR.A
						R.ASSDGFEWVSFK.T
						R.GLPLDVIQNSFDISR.E
						R.SEMTIFAPGSR.S
